# Transabdominal pre-peritoneal mesh in inguinal hernia repair in elderly: end point of our experience

**DOI:** 10.1186/1471-2482-13-S2-S24

**Published:** 2013-10-08

**Authors:** Alessia MDG Ferrarese, Stefano Enrico, Mario Solej, Alessandro Falcone, Silvia Catalano, Enrico Gibin, Silvia Marola, Alessandra Surace, Valter Martino

**Affiliations:** 1 University of Turin - Department of Oncology - School of Medicine - Teaching Hospital "San Luigi Gonzaga" - Section of General Surgery - Orbassano - Turin, Italy

## Abstract

**Background:**

Aim of this study is to present our standardized laparoscopic transabdominal preperitoneal hernia repair (TAPP) technique, and to study our experience in the elderly as far as concerns preoperative and postoperative variables.

**Methods:**

We described our standardized TAPP technique according with Stuttgart technique [1], and we evalutated our team's experience in TAPP inguinal hernia repair in elderly (> 65 yrs) and in young patients (< 65 yrs).

**Results:**

We retrospectively reviewed our Surgery Division's experience about TAPP; we included in our study 185 patients. The sample was subdivided in two groups: TAPP Group (< 65 years patients) and TAPPe Group (> 65 years patients). TAPP Group was composed by 154 patients and TAPPe Group of 31 patients. According with literature, in this subgroup recurrence rate (3,2%), early and delayed complications and mean operative time (86 min). There were no major vascular or intestinal complications. At the moment follow-up is 31 months. There were no incisional hernias on umbilical trocar. Mean satisfaction rate was excellent also in elderly patients.

**Conclusions:**

According with literature, in our experience TAPP technique is a safe and feasible procedure, even in elderly patients.

## Background

Mini-invasive approach in surgery ensures better aesthetic results, faster return to work and a lesser postoperative pain, also in elderly patients [1,2,5,7].

Aim of this study is to evaluate the feasibility of our technique in the elderly, as far as concerns preoperative and postoperative variables.

## Methods

Our work is a retrospective study conducted at University Section of General Surgery in "San Luigi Gonzaga" Hospital, Orbassano (Torino). At first we described our standardized TAPP technique.

We rewieved our experience from July 2007 to December 2012 about TAPP in elderly patients (> 65 yrs - TAPPe Group) and in young patients (< 65 yrs - TAPP Group). In our division of General Surgery the first TAPP was performed on 02/05/2007. In TAPP and TAPPe Groups were excluded patients with: important comorbidities, severe chronic obstructive pulmonary disease (COPD), previous retinal detachment, glaucoma. We also excluded patients who refused general anesthesia. We didn't excluded patients with previous abdominal surgery.

All procedures were conducted by three surgeons with more than 15 years laparoscopic experience.

We first used polypropylene high-weight meshes [3] fixed with absorbable clips, then we started to use polypropylene low-weight meshes fixed with fibrin glue [4,13] and finally we started to use self-locking Polyester meshes [12]: currently 30 TAPP (6 in elderly) were performed using this mesh, in these cases without any fixation device.

Following literature pattern, complications were divided into: recurrence (early and late), early minor complications (minor vascular lesions, seroma, scrotal hematoma), early major complications (major vascular injury, bladder injury, visceral injury, umbilical cord injury), late minor complications (chronic pain) and late major complications (incisional hernia, mesh infection, mesh rejection, intra-abdominal infection, exitus).

We finally asked patients to express an opinion from 0 to 4 as a score for their satisfaction about the TAPP procedure received.

## Surgical technique [1]

- Pneumoperitoneum in left hypochondrium by Veress needle, and access in the umbilical region by Hasson reusable trocar. Intra-abdominal pressure is maintained at 12 mmHg. Placing a disposable 5/12 mm operating trocar in the right side and a reusable 5 mm trocar in the left hip. Peritoneal incision from the anterior superior iliac spine to the median ligament. Medial dissection up to discover Cooper's ligament. Dissection on the upper side of Psoas muscle. Median dissection till complete reduction of the hernia sac and of the pre-lipomatous formation in the abdomen

- In case of bilateral defects we find it useful to proceed to the contralateral preparation before mesh placement, and we prefer to prepare two separate preperitoneal pockets

- Mesh preparation and its shaping of approximately 13 × 11 cm in the medial part, 8-9 cm in the lateral part, with median notch for the umbilical cord

- Mesh introduction and its placement in the preperitoneal pocket

- Closure of the peritoneal flap by continuous suture with Prolene 2/0 absorbable, secured with clips, after eversion of the sac in the abdomen.

## Results

From January 2007 to December 2012 in our University General Surgery division 730 hernioplasty were performed, 492 with open approach and 185 with laparoscopic approach.

Laparoscopic Group was divided into two subgroups according to the age of patients in: TAPP Group with <65 years patient (133 M, 21 F - average age 57 aa - mean BMI 26) and the TAPPe Group with >65 Years patients (29 M, 2 F - average age 69 years - mean BMI 25) (Table 1).

**Table 1:** Demographic data

	TAPP Group	TAPPe Group
Age	57 (27-87)	69 (65-87)

BMI	26	25

Sex M/F	133/ 21 (82,05% - 17,95%)	29/2 (93,5% - 6,5%)

At the time of surgery, in TAPP Group 76 defects were unilateral, 25 bilateral and 53 were recurrent hernias; in TAPPe Group 23 were unilateral hernias, 3 were bilateral and 5 were recurrent hernias (Table 2). Most defects were left hernias, in both samples. At the moment the mean follow-up is 31 months. Mean operative time was 92 minutes in TAPP group and 86 minutes in TAPPe Group (Table 3). Anatomical features were satisfactory in 83% of cases, pain's triangle and disaster's triangle were well identified in all cases.

**Table 2:** Caracteristics of defects

	TAPP Group	TAPPe Group
Unilteral hernia	76/154 (49,3%)	23/31 (74,2%)

Bilateral hernia	53/154 (34,4%)	5/31 (16,1%)

Recurrent hernia	25/154 (16,2%)	3/31 (9,7%)

**Table 3:** Intraoperative variables

	Total TAPP Group (TAPP)	Elderly TAPP Group (TAPPe)
Mean operative time (min)	92	86

Escical lesion	0	0

Majior vasular lesions	0	0

Minor vascular lesion	1	0

Nerve injury	4/154 (2,6%)	0

Umbilical cord injury	0	0

There were no major vascular injury, visceral injury or bladder injury in any case.

There were no wound infections or mesh infections in either group.

In TAPP Group 57 cases were treated using polypropylene mesh fixed with absorbable clips, 56 cases using polypropylene mesh fixed with biological glue and 24 cases using a self-locking mesh. In TAPPe Group 3 cases were treated using polypropylene mesh fixed with absorbable clips, 22 cases using polypropylene mesh secured with biological glue and 6 cases using a self-locking mesh. We never used non-absorbable metal tacks (Table 4).

**Table 4:** Caracteristics of meshes

	TAPP Group	TAPPe Group
Polprolylene mesh-non absorbable tacks	0	0

Polprolylene mesh-absorbable tacks	59 (49,3%)	3/31 (9,7%)

Polprolylene mesh-fibrin glue	66 (42,8%)	22/31 (70,9%)

Self-Locking mesh	29 (18,8%)	6/31 (19,3%)

Observed complications are as follows (Table 5,6): in TAPP group there were 4 early recurrent hernias and 2 late recurrent hernias, 1 epigastric lesion, 8 seroma and 4 chronic pain. In TAPPe Group we described only one early recurrent hernia and 2 seromas.

**Table 5:** Late complications/variables

	TAPP Group	TAPPe Group
Cronic pain	4/154 (2,6%)	0

Ventral hernia	0	0

Late recurrence	2/154 (1,29%)	0

Late reoperation rate	2/154 (1,29%)	0

**Table 6:** Early complications/variables

	TAPP Group	TAPPe Group
Total hospital stay (Mediana, gg)	1,5	1,3

Early recurrence rate	4/154 (2,6%)	1/31 (3,2%)

Early reoperation rate	4/154 (2,6%)	1/31 (3,2 %)

Seroma	8/154 (2,6 %)	0

There was no mortality. The mean patients hospital stay was: 1.5 days in TAPP Group and 1.3 days in TAPPe Group. All patients were satisfied about laparoscopic procedure (Table 7).

**Table 7:** Satisfaction score

	TAPP Group	TAPPe Group
GR 0: bad	1 (0,6%)	0

GR 1: medium	5 (3,2%)	1 (3,2%)

GR 2: good	41 (26,6%)	5 (16,1%)

GR 3: excellent	107 (69,5%)	25 (80,6%)

## Conclusions

Laparoscopic treatment of inguinal hernias is a difficult procedure that requires an adequate learning curve [1]. In our experience, operative time and hospital stay appear to be acceptable and in accord with the experience of most centres.

Short-and long-term results of the technique in terms of perioperative minor complications, post-operative pain and morbidity are in agreement with literature [7-11].

According to literature, satisfaction of patients who underwent laparoscopic procedure in our TAPP experience was excellent.

In our opinion the best short and long term perioperative results depend on careful and bloodless dissection of the preperitoneal space, meticulous reduction of the hernia sac, appropriate mesh size, its positioning and fixation; also fundamental is to completely close the peritoneal flap, leaving no gaps [1].

We consider surgery approach more difficult in the elderly in some cases [14] but we also considered laparoscopic approach is, in general, a safe and feasible technique in acute pathology [15] and a safe approach also in the elderly [16,17]. In our experience, laparoscopic repair of wound defects is a good standard technique also in the elderly.

In conclusion, in our experience, despite the retrospective study limitations, TAPP technique for inguinal hernia repair is an effective and safe technique when performed by experienced hands, also in the elderly. Perioperative intervention related morbidity appears to be within normal limits, and the superiority of laparoscopic technique in terms of post-operative discomfort, improved aesthetic results and early return to work is to be confirmed also in this type of intervention and also in the elderly.

## Competing Interests Statement

The authors declare that they have no competing interests.

## Authors' contributions

AGF: conception and design, interpretration of data, given final approval of the version to be published.

SE: conception and design, interpretration of data, given final approval of the version to be published.

MS: acquisition of data, drafting the manuscript, given final approval of the version to be published.

AF: acquisition of data, drafting the manuscript, given final approval of the version to be published.

SC: acquisition of data, drafting the manuscript, given the final approval of the version to be published.

EG: acquisition of data, drafting the manuscript, given the final approval of the version to be published.

AS: critical revision, interpretation of data, given final approval of the version to be published

VM: critical revision, interpretation of data, given final approval of the version to be published

## Declarations

Funding for this article came from personal funds.

This article has been published as part of *BMC Surgery *Volume 13 Supplement 2, 2013: Proceedings from the 26th National Congress of the Italian Society of Geriatric Surgery. The full contents of the supplement are available online at http://www.biomedcentral.com/bmcsurg/supplements/13/S2
